# Aadhar Number Marking on Surgical Plate for Forensic Identification

**DOI:** 10.7759/cureus.30060

**Published:** 2022-10-08

**Authors:** Rathod Prakash, Shreya Colvenkar, Aditya Mohan Alwala, Saideep Katkuri, MD Shakeel Ahmed

**Affiliations:** 1 Department of Oral and Maxillofacial Surgery, MNR Dental College and Hospital, Sangareddy, IND; 2 Department of Prosthodontics, MNR Dental College and Hospital, Sangareddy, IND; 3 Department of Oral and Maxillofacial surgery, MNR Dental College and Hospital, Sangareddy, IND

**Keywords:** postmortem identification, aadhar number, fixation, surgical plate, laser, forensic

## Abstract

Dental identification is the most reliable and frequently applied method of human identification, predominantly by comparing the antemortem and post-mortem records. Hence, all types of dental treatment should be recorded and kept properly. Aadhaar is a 12-digit individual identification number issued by the Unique Identification Authority of India on behalf of the Government of India to all residents of India.Marking an Aadhar number on a surgical plate can play an important role in forensic identification when other methods fail. In the case presented here, considering the importance of forensic records, the patient's surgical plates were labeled with the Aadhar number using a laser. A little effort from all dental practitioners to get surgical plates marked with patient details can help in quick identification if the need arises in the future.

## Introduction

Identification of an individual is not only important for legal and administrative purposes, but also for humanitarian reasons [1]. Proper identification is based on the comparison between known characteristics of a person with recovered characteristics from an unknown body. Identification of the deceased by the family member or close acquittance is the usual method, but this may be impossible in cases of traumatic and destructive disasters. Fingerprints are commonly used for identification in such cases, but in instances where they cannot be used, dental records could be helpful. During the reconstructive identification process, all medical and dental records gathered from an unknown victim are more reliable when compared to circumstantial evidence. Routine dental treatment records like restorations, orthodontic treatment, implants, surgical plate models, and radiographs, if properly maintained, provide a very good record [2]. Identification code having the patient's details inserted in the dentures, implants, removable and fixed orthodontic appliances as well as surgical plates helps in quick identification and brings the case to the closure [3-5]. Labeling prostheses is recommended by most dental associations and forensic odontologists worldwide [6,7].

Although several previous studies have explored labeling on removable dentures, none have concentrated on labeling surgical plates. Surgical plates and screws are used in the treatment of mandibular and maxillary fractures to provide rigid internal fixation and a functionally stable fracture site. Labeling surgical plates with identification codes using lasers will help in the quick identification of the deceased individual. Aadhaar is a 12-digit individual identification number in India, issued by the Unique Identification Authority of India on behalf of the Government of India. This article describes a case report of a patient with a symphysis fracture of the mandible, treated with open reduction and rigid internal fixation with surgical plates that were labeled with his Aadhar number.

## Case presentation

**Figure 1:**
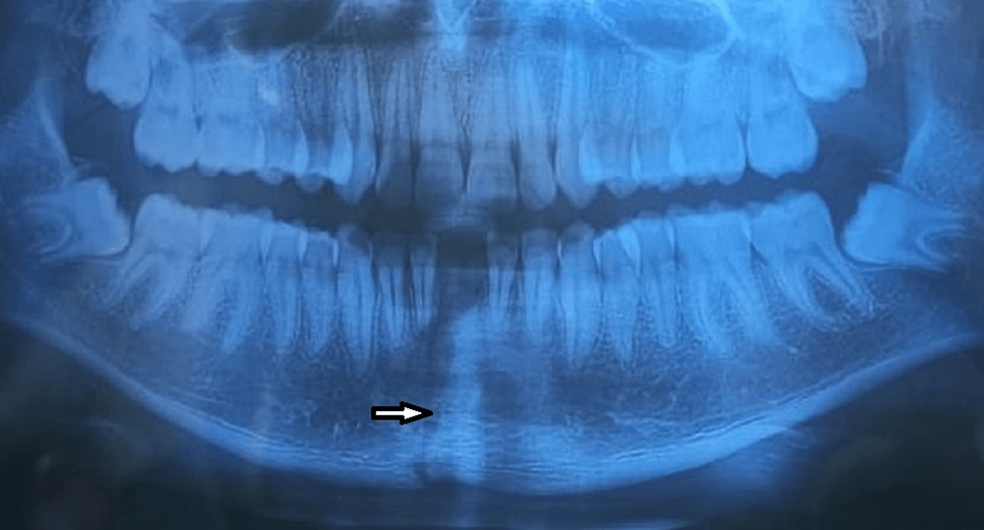
Orthopantomogram showing symphysis fracture of mandible

A 21-year-old male patient visited the department of oral and maxillofacial surgery with a symphysis fracture of the mandible caused by an accident. The orthopantomogram confirmed the presence of the symphysis fracture (Figure1).

**Figure 2:**
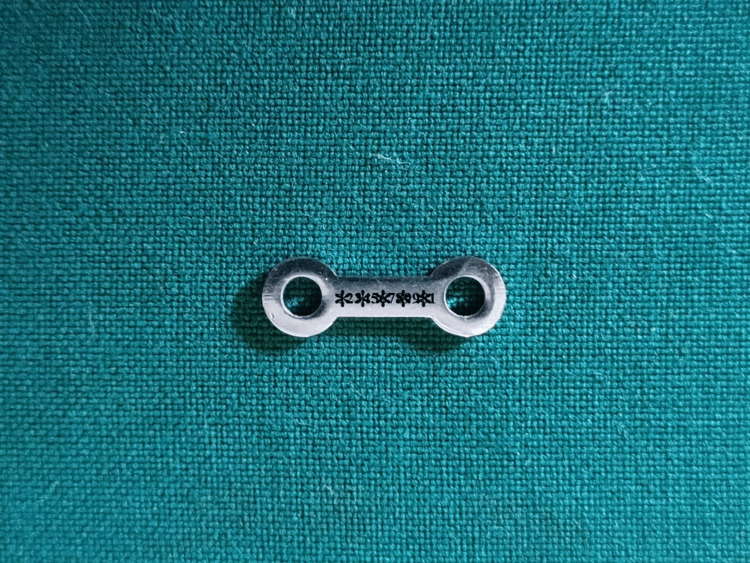
Surgical plate marked with patient's Aadhar number

Surgical plates were laser marked with the patient's Aadhar number (Figure 2).

**Figure 3:**
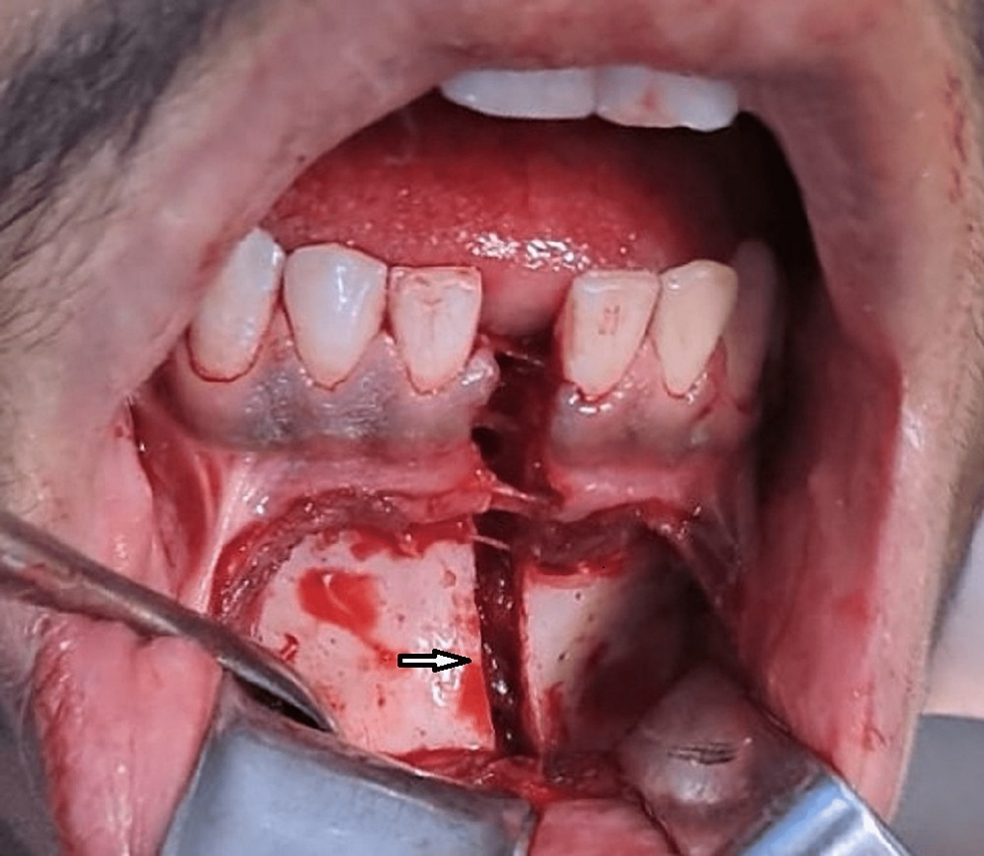
Mucoperiosteal flap reflected showing fractured site

Treatment consisted of maxillo-mandibular fixation by means of open reduction. Under local anesthesia, eyelet wire fixation and bridal wire fixation were carried out to get teeth in proper occlusion. An intraoral vestibular incision was made from canine to canine and a mucoperiosteal flap was reflected to expose the fracture site (Figure 3).

**Figure 4:**
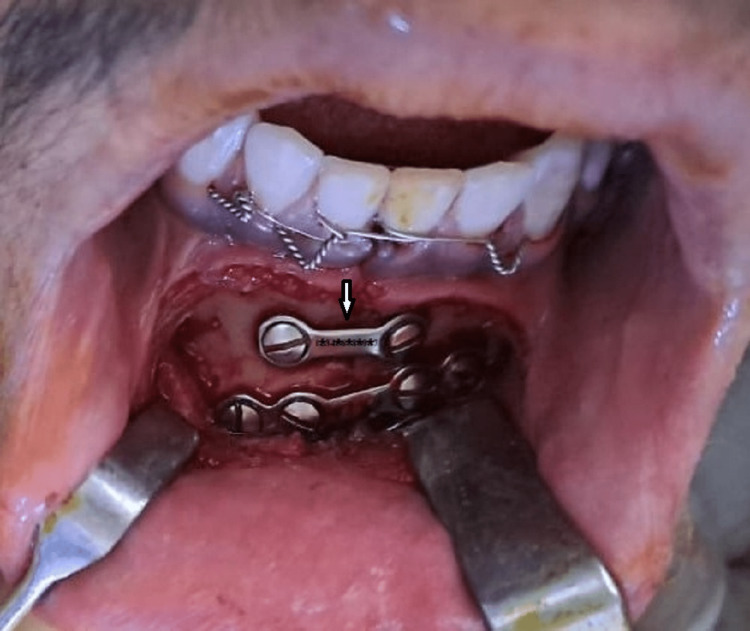
Surgical plate fixed with screws

After that rigid internal fixation under stable occlusion with a 2.5 mm four-hole stainless steel plate in the lower border of the mandible was carried out. After that, a 2.5 mm two-hole stainless steel plate with screws was placed superior to the four-hole plate (Figure 4).

The flaps were repositioned and sutured to obtain closure. The patient was prescribed medicines and followed up after three days and after a week.

## Discussion

Decedents who are severely decomposed, skeletonized, or incinerated present a challenge for identiﬁcation. Dental remains have played an important role in forensic identification when other diagnostic findings such as visual or fingerprints have been destroyed beyond recognition. [8,9]. Dental remains like dense inorganic tooth structures can withstand high temperatures during a fire, explosion, and collision compared to other body tissues and organs [10-13]. Dental surgical plates used in the treatment of fractures can play an important role in forensic identification.

The physical properties of stainless steel such as high melting point and corrosion resistance provide a good record of evaluation in forensic cases. Extreme temperatures can cause the evaporation of pulp tissue causing the tooth to split and break. Although restorative materials like amalgam and composite deteriorate at high temperatures, stainless steel can withstand temperatures up to 1,100°C thus resisting high thermal assaults [14].

The digital revolution in laser printing allows fine marking of even a few microns on the surface of surgical plates. Lasers have been used to label the cobalt-chromium dentures as well as complete dentures [15,16]. Shreya et al. described a technique where a quick response code was marked with lasers on stainless steel plate and then inserted into the denture [16]. Not all surgical plates are marked with the manufacturer code and name. Considering the importance of identification for forensic reasons, the surgical plate in the present case was labeled with the patient’s Aadhar number by laser sintering machine.

An Aadhaar number is a 12-digit, easily verifiable unique identification number that has all the patient’s information such as iris scan, fingerprints, age, and address. It is issued by the Unique Identification Authority of India to all residents of India. Priya et al. described a technique of using the Aadhar barcode as an identification mark to be inserted in the denture [17]. 

The Aadhaar card number is easily scannable by a smartphone and will give authenticated complete information about the individual in a very concise form. Most countries have a unique identification number for their citizens, which has been used for denture marking. In India, marking the surgical plate with the Aadhaar card number will serve the purpose. In the present case, the patient's Aadhar number will help in quick identification if all other methods fail.

## Conclusions

Forensic dental identification has played a very important role in natural as well as manmade disasters. Good quality dental records are an essential part of a medicolegal requirement and are important for dental identification. The patient reported with symphysis fracture of the mandible. Considering the importance of forensic records, the patient’s surgical plate was labeled with his Aadhar number. The Aadhar number is a unique identification number provided to all citizens of India. This simple marking will help in quick identification if the need arises in the future.
